
JOURNAL INFORMATION
==============================
NLM Title Abbreviation: ScientificWorldJournal
Journal ID: ScientificWorldJournal
NLM Journal ID: 101131163
Journal ID: TSWJ

The Scientific World Journal

ISSN: 2356-6140
EISSN: 1537-744X
Publisher: Wiley

ARTICLE INFORMATION
==============================
PMCID: PMC5849131
PMID: 18454250
DOI: 10.1100/tsw.2008.62
Article ID: 216938
Article version: 1
Subjects: Clinical Image

Proliferative Diabetic Retinopathy and Hemovitreous: Red Flag for Blindness

Català Jaume
Muñoz Silvia *
Hospital Universitari de Bellvitge- Ophthalmology Department, L'Hospitalet de Llobregat, Barcelona, Spain
*Silvia Muñoz: silviamq@terra.esAcademic Editor: Daniel Batlle

Publication date: 2008
Electronic publication date: 2008 Apr 20
Volume: 8
First page: 409
Last page: 410
Received 2008 Mar 25; Accepted 2008 Apr 2
Copyright: Copyright © 2008 Jaume Català and Silvia Muñoz.
Copyright year: 2008
License: This is an open access article distributed under the Creative Commons Attribution License, which permits unrestricted use, distribution, and reproduction in any medium, provided the original work is properly cited.
License URL: https://creativecommons.org/licenses/by/3.0/

Keywords: proliferative diabetic retinopathy, hemovitreous, retinal neovascularization

==============================
Clinical Image
*TheScientificWorldJOURNAL* (2008) 8, 409–410
ISSN 1537-744X; DOI 10.1100/tsw.2008.62

TheScientificWorldJOURNAL
www.thescientificworld.com

### Proliferative Diabetic Retinopathy and Hemovitreous: Red Flag for Blindness

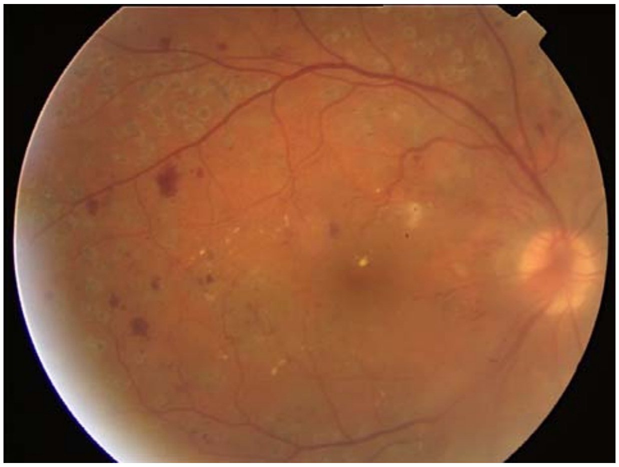

**FIGURE 1**. The scars produced by the impact of laser photocoagulation appear as grey retinal round marks surrounding the temporal vascular arcades. Multiple dot and blot hemorrhages and microaneurysms can be seen in the posterior pole. There are two types of white abnormalities: multiple lipid exudate deposition near the macula and a couple of cotton-wool spots temporal to the optic disk. An abnormal tissue emerges from the optic nerve head as well as new vessels, which cannot be properly seen because of hemovitreous.

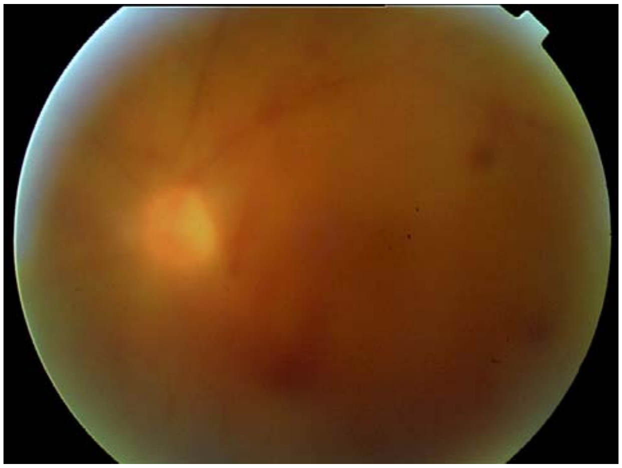

**FIGURE 2.** Dense hemovitreous obscures fundus details. Multiple retinal hemorrhages are glimpsed through media opacities.

Jaume Català and Silvia Muñoz*
*Hospital Universitari de Bellvitge- Ophthalmology Department, L'Hospitalet de Llobregat, Barcelona, Spain*

**E-mail**: silviamq@terra.es

*Received March 25, 2008; Accepted April 2, 2008; Published April 20, 2008*

**KEYWORDS:** proliferative diabetic retinopathy, hemovitreous, retinal neovascularization

### CASE 1

A 54-year-old male patient experienced sudden painless visual loss in his left eye. He had been diagnosed with diabetes mellitus 8 years prior with poor metabolic control. Diabetic retinopathy was diagnosed 2 years ago and was treated with mild scattered retinal photocoagulation.

On examination, proliferative diabetic retinopathy with optic disk neovascularization was observed in his right eye (RE) (Fig. 1). Moderate vitreous hemorrhage was detected in his left eye (LE) (Fig. 2). He underwent pars plana vitrectomy with posterior hyaloid dissection, and laser endophotocoagulation in the

*Corresponding author.
©2008 with author.
Published by TheScientificWorld; www.thescientificworld.com

LE that resolved the vitreous hemorrhage. Laser photocoagulation was scheduled to complete the treatment in the RE. Intensive metabolic control may have helped to maintain visual acuity of 20/30 in the RE and 20/25 in the LE.

### CASE 2

A 44-year-old male was seen for bilateral visual loss. He did not have ophthalmology follow-up for many years, despite having been diagnosed with diabetic retinopathy. Visual acuity was very poor. He had hand movement bilaterally. Retinal examination showed vitreous hemorrhage in both eyes that covered most of the fundus details in LE. New vessel formation was seen in the RE (Fig. 3). Bilateral proliferative diabetic retinopathy was diagnosed.

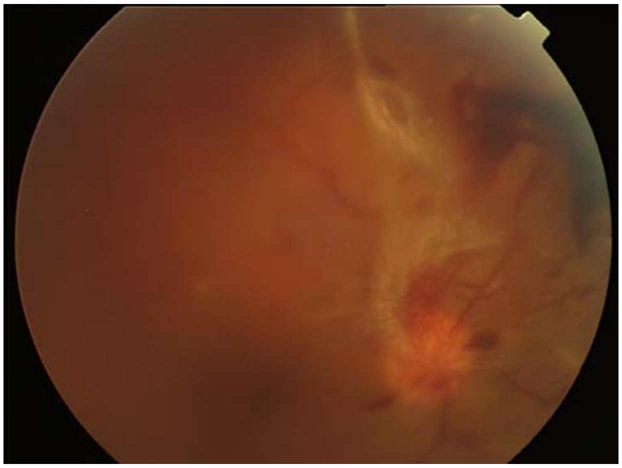

**FIGURE 3.** Case 2 RE. There is a moderate amount of blood inside the vitreous cavity. A new-vessel bunch emerges from the superior half of the optic disk accompanied by fibrous tissue at 12 o'clock. A big dark preretinal hemorrhage is seen in the superior retinal quadrant.

### REFERENCES

* Wilkinson, C.P., Ferris, F.L., Klein, R.E., et al. (2003) Proposed international clinical diabetic retinopathy and diabetic macular edema disease severity scales. *Ophthalmology* **110,** 1677–1682.
* Morello, C.M. (2007) Etiology and natural history of diabetic retinopathy: an overview. *Am. J. Health Syst. Pharm.* **64(17 Suppl 12),** S3–7.
* Liew, G. and Wang, J.J. (2007) Retinal vascular signs in diabetes and hypertension--review. *Arq. Bras. Endocrinol. Metabol.* **51(2),** 352–362.
* Dubey, A.K., Nagpal, P.N., Chawla, S., and Dubey, B. (2008) A proposed new classification for diabetic retinopathy: the concept of primary and secondary vitreopathy. *Indian J. Ophthalmol.* **56,** 23–29.

**This article should be cited as follows:**

Català, J. and Muñoz, S. (2008) Proliferative diabetic retinopathy and hemovitreous: red flag for blindness. *TheScientificWorldJOURNAL* **8**, 409–410. DOI 10.1100/tsw.2008.62.
